# Gut microbiome is associated with multiple sclerosis activity in children

**DOI:** 10.1002/acn3.51441

**Published:** 2021-08-19

**Authors:** Mary K. Horton, Kathryn McCauley, Douglas Fadrosh, Kei Fujimura, Jennifer Graves, Jayne Ness, Yolanda Wheeler, Mark P. Gorman, Leslie A. Benson, Bianca Weinstock‐Guttman, Amy Waldman, Moses Rodriguez, Jan‐Mendelt Tillema, Lauren Krupp, Anita Belman, Soe Mar, Mary Rensel, Tanuja Chitnis, Theron Charles Casper, John Rose, Janace Hart, Xiaorong Shao, Helen Tremlett, Susan V. Lynch, Lisa F. Barcellos, Emmanuelle Waubant, the U.S. Network of Pediatric MS Centers

**Affiliations:** ^1^ Division of Epidemiology University of California, Berkeley Berkeley California USA; ^2^ Department of Medicine‐ Gastroenterology University of California, San Francisco San Francisco California USA; ^3^ Department of Neurosciences University of California, San Diego La Jolla California USA; ^4^ Division of Pediatric Neurology University of Alabama Birmingham Alabama USA; ^5^ Department of Neurology Boston Children’s Hospital Boston Massachusetts USA; ^6^ Department of Neurology State University of New York Buffalo New York USA; ^7^ Department of Neurology Children’s Hospital of Philadelphia Philadelphia Pennsylvania USA; ^8^ Department of Neurology Mayo Clinic Rochester Minnesota USA; ^9^ Pediatric Multiple Sclerosis Center New York University Langone Medical Center New York New York USA; ^10^ Department of Neurology Washington University in St. Louis St. Louis Missouri USA; ^11^ Department of Neurology Cleveland Clinic Cleveland Ohio USA; ^12^ Division of Child Neurology Massachusetts General Hospital Boston Massachusetts USA; ^13^ School of Medicine University of Utah School Salt Lake City Utah USA; ^14^ Department of Neurology University of California, San Francisco San Francisco California USA; ^15^ Department of Medicine University of British Columbia Vancouver British Columbia Canada

## Abstract

**Objective:**

To identify features of the gut microbiome associated with multiple sclerosis activity over time.

**Methods:**

We used 16S ribosomal RNA sequencing from stool of 55 recently diagnosed pediatric‐onset multiple sclerosis patients. Microbiome features included the abundance of individual microbes and networks identified from weighted genetic correlation network analyses. Prentice‐Williams‐Peterson Cox proportional hazards models estimated the associations between features and three disease activity outcomes: clinical relapses and both new/enlarging T2 lesions and new gadolinium‐enhancing lesions on brain MRI. Analyses were adjusted for age, sex, and disease‐modifying therapies.

**Results:**

Participants were followed, on average, 2.1 years. Five microbes were nominally associated with all three disease activity outcomes after multiple testing correction. These included butyrate producers *Odoribacter* (relapse hazard ratio = 0.46, 95% confidence interval: 0.24, 0.88) and *Butyricicoccus* (relapse hazard ratio = 0.49, 95% confidence interval: 0.28, 0.88). Two networks of co‐occurring gut microbes were significantly associated with a higher hazard of both MRI outcomes (gadolinium‐enhancing lesion hazard ratios (95% confidence intervals) for Modules 32 and 33 were 1.29 (1.08, 1.54) and 1.42 (1.18, 1.71), respectively; T2 lesion hazard ratios (95% confidence intervals) for Modules 32 and 33 were 1.34 (1.15, 1.56) and 1.41 (1.21, 1.64), respectively). Metagenomic predictions of these networks demonstrated enrichment for amino acid biosynthesis pathways.

**Interpretation:**

Both individual and networks of gut microbes were associated with longitudinal multiple sclerosis activity. Known functions and metagenomic predictions of these microbes suggest the important role of butyrate and amino acid biosynthesis pathways. This provides strong support for future development of personalized microbiome interventions to modify multiple sclerosis disease activity.

## Introduction

Multiple sclerosis (MS) is a chronic, inflammatory disease of the central nervous system with symptoms and disease activity that vary greatly from person to person. Despite recent advances in treatment, there is no cure for MS, and it remains largely unknown what factors contribute to disease activity over time. Smoking, obesity, Epstein–Barr virus infection, low vitamin D, and over 200 genetic variants are established risk factors for developing MS. However, with the exception of low vitamin D, they have not been convincingly or consistently shown to contribute to MS outcomes such as clinical relapse or lesion activity on brain MRI.^1^, ^2^ Thus, it is critical to investigate the novel drivers of MS activity that might inform interventions designed to attenuate disease course.

Recently, a growing body of experimental and observational studies have suggested that microbes in the gut contribute to MS pathogenesis.^3^ Several potential biological mechanisms include direct and indirect interactions of microbes and microbial metabolites with immune cells and pro‐inflammatory chemokines and cytokines, all of which can influence the central nervous system.^4^, ^5^, ^6^ However, it remains unknown which, if any, features of the gut microbiome contribute to disease activity in MS. In animal models of MS, a germ‐free environment has been associated with lower disease activity, and perturbations to the gut microbiota have been associated with changes in disease activity.^7^, ^8^, ^9^ Additionally, the oral administration of *Bacteroides fragilis* has been associated with lower “clinical” scores in relapsing mouse models of MS.^10^ One small observational study of the gut microbiome and disease activity in persons with MS investigated clinical relapse as the outcome.^11^ After adjusting for age and disease‐modifying therapy (DMT) use, the relative absence of *Fusobacteria* was associated with a higher chance of relapse (hazard ratio = 3.2; 95% confidence interval: 1.2, 9.0). This study was limited in size, did not investigate the role of specific microbial taxa (such as genus or species) or co‐occurring networks of microbes, and did not include other clinical outcomes. No studies have investigated the association between gut microbes and direct measures of disease activity assessed by brain MRI, which is sensitive to lesion formation, more common than clinical relapses,^12^ and can serve as a biomarker of active inflammation.

In this study, we utilized 16S ribosomal RNA sequencing profiles from the stool of 55 pediatric‐onset MS cases to investigate whether specific features of the gut microbiome were associated with time to three separate disease activity outcomes: clinical relapses, new gadolinium‐enhancing lesions (representing areas of active inflammation), and new or enlarging T2‐hyperintense lesions (markers of overall disease burden). Using pediatric‐onset cases (individuals with MS symptom onset before 18 years of age) to investigate these associations was advantageous, because symptom onset was likely closer to the biological onset of disease. Further, children and youth have higher disease activity, compared to adults, making it more feasible to study relapses and MRI activity over time.^13^, ^14^

## Participants and methods

### Study population

Between 2012 and 2018, 60 individuals with MS onset before 18 years old were enrolled and provided stool samples that could be analyzed. Six enrollees did not have clinical follow‐up (leaving 54 in the “clinical cohort”) and 14 did not have subsequent MRI scan data available (leaving 46 in the “MRI cohort”). Participants were recruited from seven sites in the US Network of Pediatric MS Centers including the University of California San Francisco, State University of New York at Buffalo, University of Alabama at Birmingham, Boston Children’s Hospital, Stony Brook University Medical Center, Children’s Hospital of Philadelphia, and New York University. At stool sample collection (“baseline”), all participants were within 24 months of symptom onset and met the 2010 McDonald criteria for MS.^15^ Exclusion criteria included: participant’s banked serum tested positive for myelin oligodendrocyte glycoprotein antibodies, participant had been exposed to a systemic antibiotic, probiotic, or steroid within 1 month prior to stool sample collection, or participant had previously used a cytotoxic immunosuppressant.

All parents and participants provided written informed consent and assent. Ethical approval for the study was obtained from each institution’s Institutional Review Board.

### Clinical relapses and MRI outcomes

During the study period, participants were seen for regular care at the enrolling clinic, which usually included a visit every 6 months, with additional visits if the participant experienced a relapse or other clinical reason. MRI scans were ordered at study visits (per the primary neurologist) and conducted using each site’s scanner and local protocol. Data from follow‐up visits (including the dates of relapse onset, use of DMTs, and MRI) were prospectively entered into a web‐based registry. The Data Coordinating and Analysis Center at the University of Utah managed the data and performed quality control.

Three outcomes, which could recur over the study period, were assessed separately: clinical relapse(s), development of new gadolinium‐enhancing brain lesion(s), and development of new or enlarging T2 hyperintense brain lesion(s). These outcomes were defined previously.^16^ We considered a lesion new or enlarging relative to the previous MRI.

### Gut microbiota profiling

A parent collected the participant's first stool of the day and shipped overnight on ice to the University of California, San Francisco, where it was stored at −80°C before processing. DNA was extracted, and the V4 region of the 16S rRNA gene was amplified for sequencing, as previously described.^17^

Forward and reverse reads were processed separately, and quality filtered using the DADA2 package version 1.9.0. in R.3.5.2.^18^, ^19^ Reads having more than two expected errors or ≤150 base pairs in length were removed. Error rates of the filtered dereplicated reads were estimated using 100,000 sequences. Paired sequencing reads with a minimum overlap of 25 base pairs were merged to obtain the full denoised sequences. Chimeras and any sequences abnormally short or long were removed. Amplicon sequence variants (ASVs) were inferred exactly, resolving variants that differ by as little as one nucleotide. Taxonomy was assigned using the naïve Bayesian classifier method (Kingdom to Family) and exact string matching (Genus and Species) utilizing the SILVA v132 reference database.^18^, ^20^, ^21^ It is important to note that while an ASV has a unique nucleotide sequence, it might not be assigned a unique species or taxonomy due to limitations of 16S sequencing in determining strain‐level differences among species and missing microbial genomes in reference databases. Using the *decontam* package, ASVs with a contaminant classification threshold *p* < 0.1 were removed.^22^ ASVs containing less than 1/1000th of a percent of total reads were removed. Sequencing reads were representatively rarefied to the minimum sequencing depth (84,818 reads/sample) 100 times, and the rarefied sample profile closest to the sample‐specific centroid was selected, as described previously.^17^ The resulting tables included 1,482 ASVs.

### Covariates

Upon enrollment, participants completed a questionnaire including age, symptom onset, race, ethnicity, and sex. Medication history was obtained, and subsequent medication use was tracked over the follow‐up period. Disease‐modifying therapies included those previously described.^16^ For relapse‐related analyses, time‐varying DMT use was defined as “yes” if the subject used a DMT within 3 months prior to the respective relapse, and “no” if otherwise. For MRI analyses, time‐varying DMT use was defined as “yes” if the subject was using a DMT during the period between the respective MRI and the previous MRI and “no” if otherwise.

### Statistical analyses

#### Alpha and beta diversity

All statistical analyses were completed using R and the *phyloseq* package.^23^ Alpha (within sample) diversity was evaluated using a rarefied ASV table with richness (Chao1 and Faith’s phylogenetic diversity) and evenness (Pielou) estimators. To test for the association between each alpha diversity metric and time to each disease activity outcome, we used Prentice‐Williams‐Petersen time‐to‐event models.^24^ These are an extension of Cox proportional hazard models and are appropriate for outcomes that can recur over the study period and are not independent.

**Figure 1:**
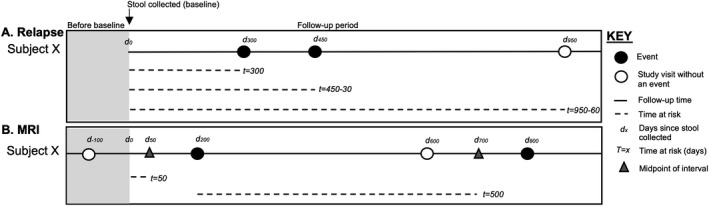
Example of survival analyses for relapse and MRI outcomes. (A) For relapse analyses, time to each relapse (an “event” in panel A) started on the day stool was collected (d_0_) and ended on the day of each respective relapse or the last study visit where relapse status was known (d_x_). A 30‐day period was subtracted from the at‐risk period following a relapse because, by definition, a new relapse must be at least 30 days after the previous. (B) For MRI analyses, an “event” was defined as a brain MRI that indicated a new or enlarging lesion compared to the prior MRI. Specifically, we used two MRI outcomes considered separately: new gadolinium‐enhancing lesion and new or enlarging T2 lesion. Because the timing of MRI varies in clinical practice and the specific time of lesion activity is unknown, midpoint survival analyses were used. For the first MRI event after stool was collected, time at risk started on the date stool was collected and ended on the midpoint between the first MRI event and the prior MRI where an event did not occur (or the MRI that preceded baseline). For subsequent MRI events, time at risk started on the date of the previous MRI with an event and ended on the midpoint between the respective MRI event and the prior MRI where an event did or did not occur. Individuals were censored at the date of their last MRI (with or without an MRI event) before the study end.

For relapse analyses, clinical cohort members were followed from baseline to the earlier of the final clinic visit or occurrence of a third relapse (Fig. 1 panel A). Relapses were truncated after the first three to prevent the estimation of hazard ratios (HRs) in event strata with few individuals. Time to each relapse (or final clinic visit) was defined as the total time from baseline to each respective event. Because, by definition, a new relapse cannot occur until at least 30 days after the previous relapse, a 30‐day period was discounted from the follow‐up time at risk for each subsequent relapse.

For brain MRI analyses, MRI cohort members were followed from baseline to the earlier of the final MRI or occurrence of a second new/enlarging lesion (gadolinium‐enhancing and T2 lesions were evaluated separately) (Fig. 1 panel B). Data were truncated after the first two new/enlarging lesions. Since a new or enlarging lesion was relative to a past MRI, we defined a “baseline MRI” as the MRI that occurred closest, but previously, to stool sample collection. Time to each new/enlarging lesion (or final MRI visit) was defined as the gap time between a new/enlarging lesion and the previous new/enlarging lesion (or baseline if first new/enlarging lesion). We used the midpoint of time between a MRI with a new/enlarging lesion and prior MRI (with or without new/enlarging lesion) as an estimate of when the new lesion developed.^16^, ^25^ Between baseline and a new/enlarging lesion, the midpoint was halfway between the baseline MRI and the new/enlarging lesion, and time between stool collection and the midpoint was used for the respective at‐risk interval. If the midpoint between the baseline MRI and first MRI with a new/enlarging lesion after baseline occurred before stool was collected, it was excluded from analyses.

For all Prentice‐Williams‐Petersen models, robust variance was computed, and HRs and 95% confidence intervals (CIs) were estimated for each alpha diversity metric and each MS activity outcome, adjusting for sex, age at event, and DMT use. The proportional hazard assumption for each model was assessed using the *cox.zph* function in the *Survival* package. All alpha diversity metrics met the proportional hazard assumption.

For beta (between sample) diversity, weighted and unweighted UniFrac distance matrices were constructed.^26^ Relationships between each beta diversity metric and whether a participant had a clinically meaningful relapse rate (annual rate ≥0.5, i.e., more than one relapse over 2 years), had any new gadolinium‐enhancing lesions, or had any new or enlarging T2 hyperintense lesions over the follow‐up period were assessed using permutational multivariate analysis of variance (PERMANOVA) using *adonis2*. Models were adjusted for sex, age at stool collection, and whether a participant was using a DMT when stool was collected.

#### ASV‐level relative abundance

To identify whether specific gut microbes were associated with subsequent disease activity, we used Prentice‐Williams‐Petersen models described above to estimate HRs and 95% CIs for each ASV and each disease activity outcome, adjusted for age, sex, and DMT use. ASVs identified in <20% of a respective analytic cohort (clinical or MRI) were excluded to reduce potentially spurious taxa and reduce the burden of multiple testing with a small sample. Rarefied counts of each ASV were dichotomized according to prevalence. ASVs in 20% to <80% of samples were categorized as “present” or “absent” if any or no taxa reads were in the sample. ASVs in ≥80% of samples were categorized as “high” or “low” depending on whether samples had ≥ or < the median number of taxa reads. This resulted in 271 ASVs available for individual‐level analyses for the clinical cohort and 256 ASVs for the MRI cohort. For each disease activity outcome, observations were corrected for false discovery rate (FDR) using the Benjamini–Hochberg method.^27^ ASVs with FDR *q‐value*<0.05 were considered significant.^27^ The proportional hazard assumption for significant ASVs was assessed the same as above, and all met the proportional hazard assumption.

#### Microbial network analysis

The co‐occurrence networks of ASVs in at least 10% of samples (resulting in 437 ASVs available for the clinical cohort and 426 for the MRI cohort) were constructed from an unrarefied ASV table using *SPIEC‐EASI* and *WGCNA* packages.^28^, ^29^ A correlation matrix was generated using *SPIEC‐EASI*, transformed to an adjacency matrix using soft thresholding, and a topology overlap matrix was generated. The topology overlap matrix was hierarchically clustered using *hclust*, and the resulting dendrogram was cut using *dynamicTreeCut* in the *stats* package to generate modules (clusters). Modules needing at least three ASVs to be retained. Correlated modules (*r* ≥ 0.5) were combined, generating a dissimilarity matrix for further hierarchical clustering. The quantitative values of each module were calculated for each participant from module eigengenes, defined as the first principal component of the abundance matrix of a respective module. Each module eigengene was tested for its association with time to relapse, new gadolinium‐enhancing lesions, and new or enlarging T2 hyperintense lesions using the Prentice‐Williams‐Petersen models described above, adjusting for age, sex, and DMT use. To improve the interpretability of results, we presented beta coefficients and HRs from regression coefficients and 95% CIs scaled to a 0.1‐unit increase in module eigengenes. Analyses were corrected for FDR, and modules with an FDR *q*‐value<0.05 were considered significant. The proportional hazard assumption for significant ASVs was assessed the same as above. One significant module did not meet the proportional hazard assumption, so a time by eigengene interaction term was added to the model. The interaction term did not have *p* < 0.05, so the HR and 95% CI for the module eigengene from the noninteraction term model were presented.

#### Metagenomic prediction

Conserved functional genes of microbes within each significant module were predicted using PICRUSt 2.^30^ For each significant module, predicted gene counts were grouped into MetaCyc metabolic pathways.^31^ We estimated the association between predicted metabolic pathways in at least 20% of samples and the disease activity outcome(s) previously identified as associated with the respective module. Pathway abundances were dichotomized as > or ≤ the respective pathway’s median abundance. HRs and 95% CIs were estimated using Prentice‐Williams‐Petersen models, adjusted for age, sex, and disease‐modifying use, and corrected for FDR.

### Data availability

The data that support the findings of this study are available on request from the corresponding author.

## Results

### Characteristics of pediatric‐onset multiple sclerosis microbiome cohort

Among all 55 cohort members, the average age at baseline was 15.9 years (IQR = 2.5), 72.7% were female, 67.2% identified as white, and 36.3% identified as Hispanic (Table 1). The distribution of these characteristics match the sex, age, race, and ethnicity distribution of pediatric‐onset MS in the United States.^32^ Approximately half were using a DMT at baseline, of which 25.0% were using interferon beta and 64.3% were using glatiramer acetate. The proportion of individual’s ASVs belonging to a particular taxonomic class did not significantly differ by baseline DMT use categories (none, glatiramer acetate, interferon beta, or other DMT), except for Melainabacteria (*p* = 0.0002) and Verrucomicrobiae (*p* = 0.049) (data not shown, Supplementary Fig. S1). Among all 55 participants, 54 were prospectively followed and evaluated for the presence (or absence) of clinical relapses (“clinical cohort”) and 46 had at least one MRI scan available (“MRI cohort”).

**Table 1:** Cohort characteristics of pediatric‐onset multiple sclerosis cases at baseline and during follow‐up.

Characteristics	Combined cohorts	Clinical cohort	MRI cohort
Baseline (stool sample collection)			
N (%)	55 (100.0)	54 (98.2)	46 (83.6)
Age (mean, IQR)	15.9 (2.5)	15.9 (2.5)	15.8 (2.6)
Age at disease onset, years (mean, IQR)	14.7 (2.7)	14.7 (2.8)	14.6 (2.7)
Sex (female) (n, %)	40 (72.7)	39 (72.2)	33 (89.2)
Race (n, %)			
Asian	4 (7.3)	4 (7.41)	3 (6.52)
Black	6 (10.9)	6 (11.1)	6 (13.0)
White	37 (67.2)	36 (66.7)	32 (69.6)
Other	6 (10.9)	6 (11.1)	5 (10.9)
Not reported	2 (3.6)	2 (3.7)	0 (0.0)
Hispanic (n, %)	20 (36.3)	19 (35.2)	16 (34.8)
Expanded Disability Status Scale (mean, IQR)	1.1 (1.5)	1.1 (1.5)	1.2 (1.3)
Disease‐modifying therapy exposed (n, %)	28 (50.9)	28 (51.9)	24 (52.2)
Interferon beta (n, %)	7 (25.0)	7 (25.0)	6 (25.0)
Glatiramer acetate (n, %)	18 (64.3)	18 (64.3)	16 (66.7)
Over follow‐up period for the clinical cohort			
Follow‐up time after stool collection, years (mean, IQR)		2.4 (2.1)	
Experienced relapse over follow‐up period (n, %)		24 (44.4)	
Number of relapses (mean, IQR)		0.9 (2.0)	
Time to first relapse after stool collection, days (mean, IQR)		297.8 (458.5)	
Relapse preceded by DMT use within 90 days prior (n, %)		37 (75.5)	
Over follow‐up period for the MRI cohort			
Time between baseline MRI and stool collection, days (mean, IQR)			89.2 (70.8)
Time to first MRI after stool collection, days (mean, IQR)			189.9 (184.0)
Gadolinium‐enhancing lesions:			
Follow‐up time, years (mean, IQR)			2.0 (1.7)
Had a new lesion over follow‐up period (n, %)			17 (40.0)
Number of new lesions (mean, IQR)			1.4 (1.0)
DMT used between MRI with new lesion and prior MRI (n, %)			52 (92.9)
T2 hyperintense lesions:			
Follow‐up time, years (mean, IQR)			1.9 (1.5)
Had a new/enlarging lesion over follow‐up period (n, %)			25 (54.3)
Number of new/enlarging lesions (mean, IQR)			1.4 (1.0)
DMT used between MRI with new/enlarging lesion and prior MRI (n, %)			33 (97.1)

Abbreviations: DMT, disease‐modifying therapy; IQR, interquartile range.

The characteristics of these cohorts were similar, albeit a higher proportion of girls were in the clinical cohort relative to MRI cohort (Table 1). For the relapse analyses, participants were followed for an average of 2.4 years (IQR = 2.1) after baseline during which time 44.4% experienced a relapse. Of the relapses that occurred, 75.5% were using a DMT in the 3 months prior. Participants were followed for a similar amount of time for the gadolinium‐enhancing (mean = 2.0 years, IQR = 1.7) and T2 hyperintense lesion (mean = 1.9 years, IQR = 15) analyses. Over the follow‐up period, approximately half of participants had a new or enlarging T2 hyperintense lesion (54.3%) while 40.0% had a new gadolinium‐enhancing lesion.

### Gut microbiome alpha and beta diversities were not associated with multiple sclerosis activity

**Figure 2:**
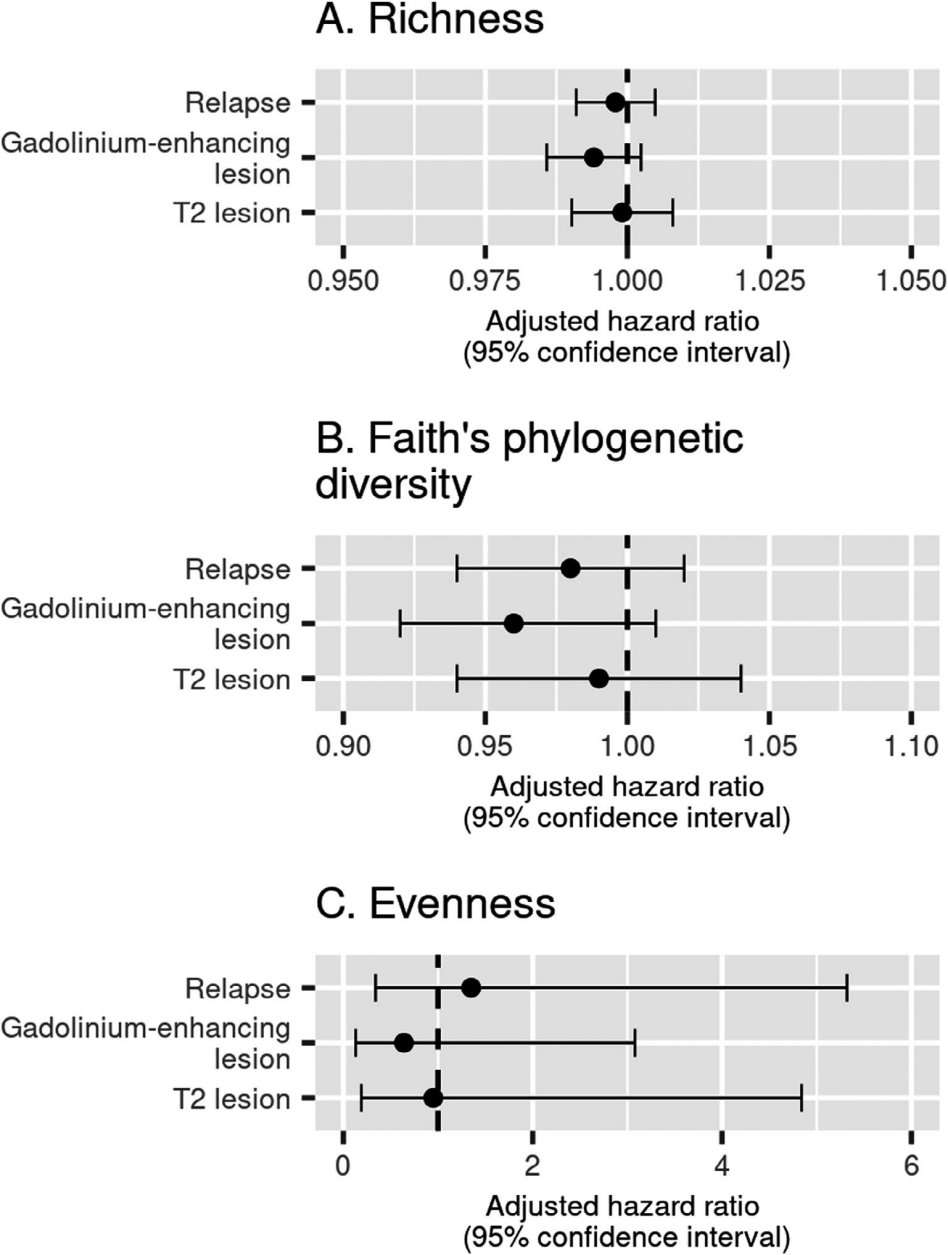
Microbial alpha diversity was not associated with clinical relapses or MRI outcomes in pediatric‐onset multiple sclerosis. (A) The Chao1 microbial richness estimator was not associated with relapse (HR = 1.00; 95% CI: 0.99, 1.00; *p* = 0.56), new gadolinium‐enhancing lesion on MRI (HR = 0.99; 95% CI: 0.99, 1.00; *p* = 0.16), or new or enlarging T2 hyperintense lesion on MRI (HR = 1.00; 95% CI: 0.99, 1.01; *p* = 0.84). (B) The Faith’s phylogenetic diversity microbial richness estimator was not associated with relapse (HR = 0.98; 95% CI: 0.94, 1.02; *p* = 0.29), new gadolinium‐enhancing lesion on MRI (HR = 0.96; 95% CI: 0.92, 1.01; *p* = 0.15), or new or enlarging T2 hyperintense lesion on MRI (HR = 0.99; 95% CI: 0.94, 1.04; *p* = 0.77). (C) Microbial evenness (Pielou estimator) was not associated with relapse (HR = 1.35; 95% CI: 0.34, 5.32; *p* = 0.67), new gadolinium‐enhancing lesions on MRI (HR = 0.64; 95% CI: 0.13, 3.08; *p* = 0.58), or new or enlarging T2 hyperintense lesions on MRI (HR = 0.95; 95% CI: 0.19, 4.84; *p* = 0.95). Beta coefficients and related HRs and 95% CIs for evenness were scaled to represent a 0.1‐unit change in evenness. Regression models adjusted for sex, age, and disease‐modifying therapy use.

**Figure 3:**
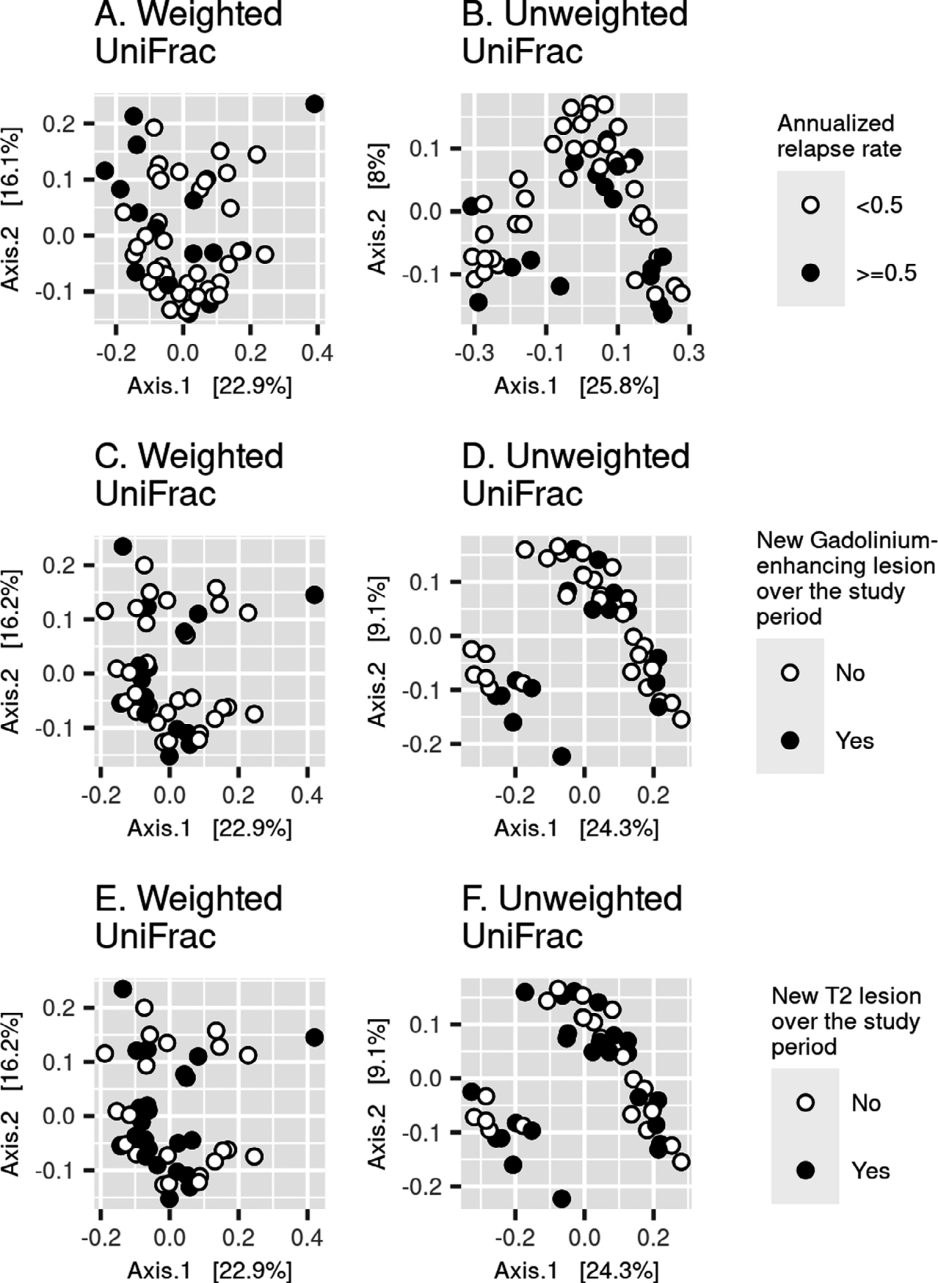
Variance in fecal microbiota composition was not associated with pediatric‐onset multiple sclerosis clinical relapse and MRI outcomes. Having, on average, more than 0.5 relapses per year was not associated with beta diversity using (A) weighted UniFrac (PERMANOVA *R^2^* = 0.01, *p* = 0.78) or (B) unweighted UniFrac distance matrices (PERMANOVA *R^2^* = 0.02, *p* = 0.38). Having any new gadolinium‐enhancing lesions over the study period was not associated with beta diversity using (C) weighted UniFrac (PERMANOVA *R^2^* = 0.01, *p* = 0.78) or (D) unweighted UniFrac distance matrices (PERMANOVA *R^2^* = 0.02, *p* = 0.42). Having any new or enlarging T2 hyperintense lesions over the study period was not associated with beta diversity using (E) weighted UniFrac (PERMANOVA *R^2^* = 0.02, *p* = 0.43) or (F) unweighted UniFrac distance matrices (PERMANOVA *R^2^* = 0.02, *p* = 0.63). PERMANOVA models adjusted for sex, age, and disease‐modifying therapy use. The first two principal coordinates from principal coordinate analysis were plotted.

Alpha diversity was not significantly associated with relapse (*p_chao1_* = 0.56, *p_faith_* = 0.29, *p_evenness_* = 0.67), new gadolinium‐enhancing lesions (*p_chao1_* = 0.16, *p_faith_* = 0.15, *p_evenness_* = 0.58), or new or enlarging T2 hyperintense lesions (*p_chao1_* = 0.84, *p_faith_* = 0.77, *p_evenness_* = 0.95) (Fig. 2). For beta diversity, relapse and MRI outcomes did not explain the observed variance in microbiota composition in fecal samples (Fig. 3). Over the study period, irrespective of whether a weighted or unweighted UniFrac distance matrix was employed, variance in fecal microbiota composition was not related to MS activity outcomes: annualized relapse rate ≥0.5 (weighted UniFrac PERMANOVA *R^2^* = 0.01, *p* = 0.78; unweighted UniFrac PERMANOVA *R^2^* = 0.02, *p* = 0.38), having any new gadolinium‐enhancing lesions (weighted UniFrac PERMANOVA *R^2^* = 0.01, *p* = 0.78; unweighted UniFrac PERMANOVA *R^2^* = 0.02, *p* = 0.42), or having any new T2 hyperintense lesions (weighted UniFrac PERMANOVA *R^2^* = 0.02, *p* = 0.43; unweighted UniFrac PERMANOVA *R^2^* = 0.02, = 0.63).

### Five gut microbes were nominally associated with all three multiple sclerosis activity outcomes

**Figure 4:**
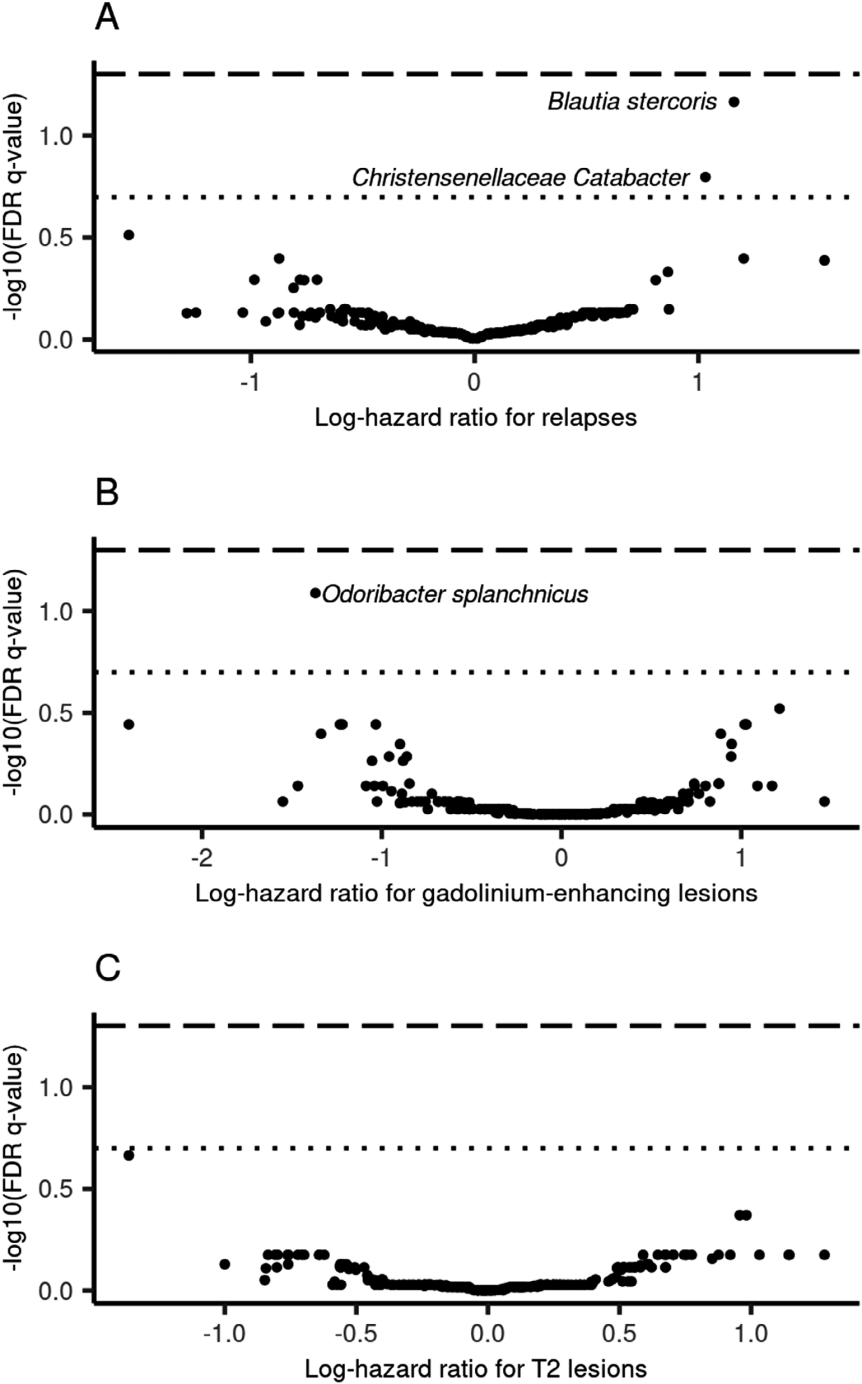
No species of gut microbes were significantly (FDR *q* < 0.05) associated with pediatric‐onset multiple sclerosis activity outcomes. The long‐dashed line indicated an FDR *q* cutoff of 0.05 and the small dotted line indicated a less conservative threshold FDR *q =* 0.2. Each point was an ASV. The genus and species (or lowest known taxonomy) of ASVs associated with a respective outcome with FDR *q* < 0.2 were labeled. Regression models were adjusted for sex, age, and disease‐modifying therapy use. (A) Adjusted log‐hazard ratios for relapse; (B) adjusted log‐hazard ratios for new gadolinium‐enhancing lesions on MRI; and (C) adjusted log‐hazard ratios for new or enlarging T2 hyperintense lesions on MRI.

A lack of relationship between MS activity and variance in overall fecal microbiota composition does not preclude the possibility that specific microbes may contribute to MS pathogenesis. For this reason, we tested whether specific ASVs were associated with pediatric‐onset MS outcomes. No ASVs were significantly associated with disease activity outcomes using a conservative threshold of FDR *q* < 0.05 (Fig. 4, see Supplementary Table S1 for full results). Using a less stringent cutoff of FDR *q* < 0.2, we identified three ASVs associated with disease activity. Two of these were associated with higher hazard of relapse: *Blautia stercoris* (HR: 3.19, 95% CI: 1.72, 5.92) and an unidentified species within the genus *Catabacter* (HR: 2.81, 95% CI: 1.51, 5.22). One ASV was associated with a lower hazard of new gadolinium‐enhancing lesions, *Odoribacter splanchnicus* (HR: 0.25, 95% CI: 0.12, 0.54).

**Figure 5:**
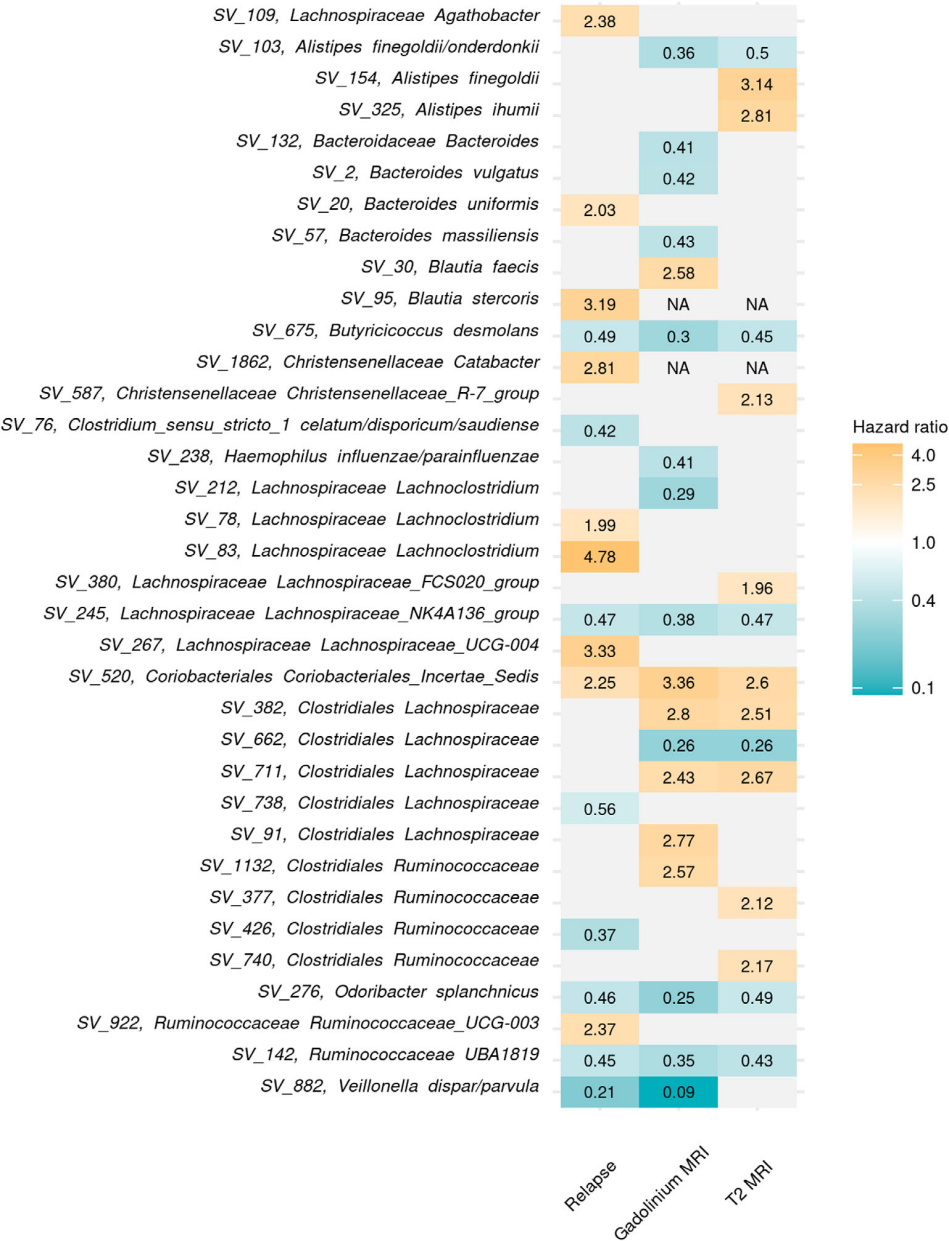
Five gut microbes were associated with all three pediatric‐onset multiple sclerosis activity outcomes. Each row was an ASV that was associated with either relapse, new gadolinium‐enhancing lesions, or new or enlarging T2 hyperintense lesions at *p* < 0.05. Hazard ratios, adjusted for sex, age, and disease‐modifying therapy use, were shown for each significant (*p* < 0.05) ASV‐outcome association. Gray indicated an ASV–outcome association was not significant. “NA” indicated an association was not estimated because the ASV was not in at least 20% of the respective sample. Rows were labeled with ASV ID and the lowest known taxonomic classification. Rows were arranged by taxonomic *order*.

To explore whether there may be microbes associated with all three disease activity outcomes, we compared the effect sizes of ASVs across all three outcomes if the ASV was associated with at least one outcome at *p* < 0.05. While several ASVs were not tested in both the relapse and MRI analyses because they were not in at least 20% of both samples, we identified five ASVs associated with all three disease activity outcomes (Fig. 5). Four of these showed protective effects across all outcomes, meaning having any of the respective ASV (or above the median number of reads) was associated with a lower hazard of relapses, gadolinium‐enhancing lesions, and T2 hyperintense lesions. These included *Butyricicoccus desmolans* (HR_relapse_ = 0.49, 95% CI: 0.28, 0.88), *Odoribacter splanchnicus* (HR_relapse_ = 0.46, 95% CI: 0.24, 0.88), an unidentified species in the *Lachnospiraceae NK4A136* group (HR_relapse_ = 0.47, 95% CI: 0.24, 0.89), and *Ruminococcaceae* (HR_relapse_ = 0.45, 95% CI: 0.22, 0.91). For these ASVs, similar HRs were observed for MRI outcomes (Fig. 5 and Supplementary Table S1). In contrast, having any reads of SV_520, an unspecified member of *Coriobacteriales* was associated with more than double the hazard for all three disease activity outcomes (HR_relapse_ = 2.25, 95% CI: 1.12, 4.49; HR_Gad_ = 3.36, 95% CI: 1.54, 7.35; HR_T2 =_ 2.60, 95% CI: 1.34, 5.08). The abundance of each of these five ASVs did not significantly differ by baseline DMT status (data not shown).

### Gut microbial networks were associated with MRI outcomes

**Figure 6:**
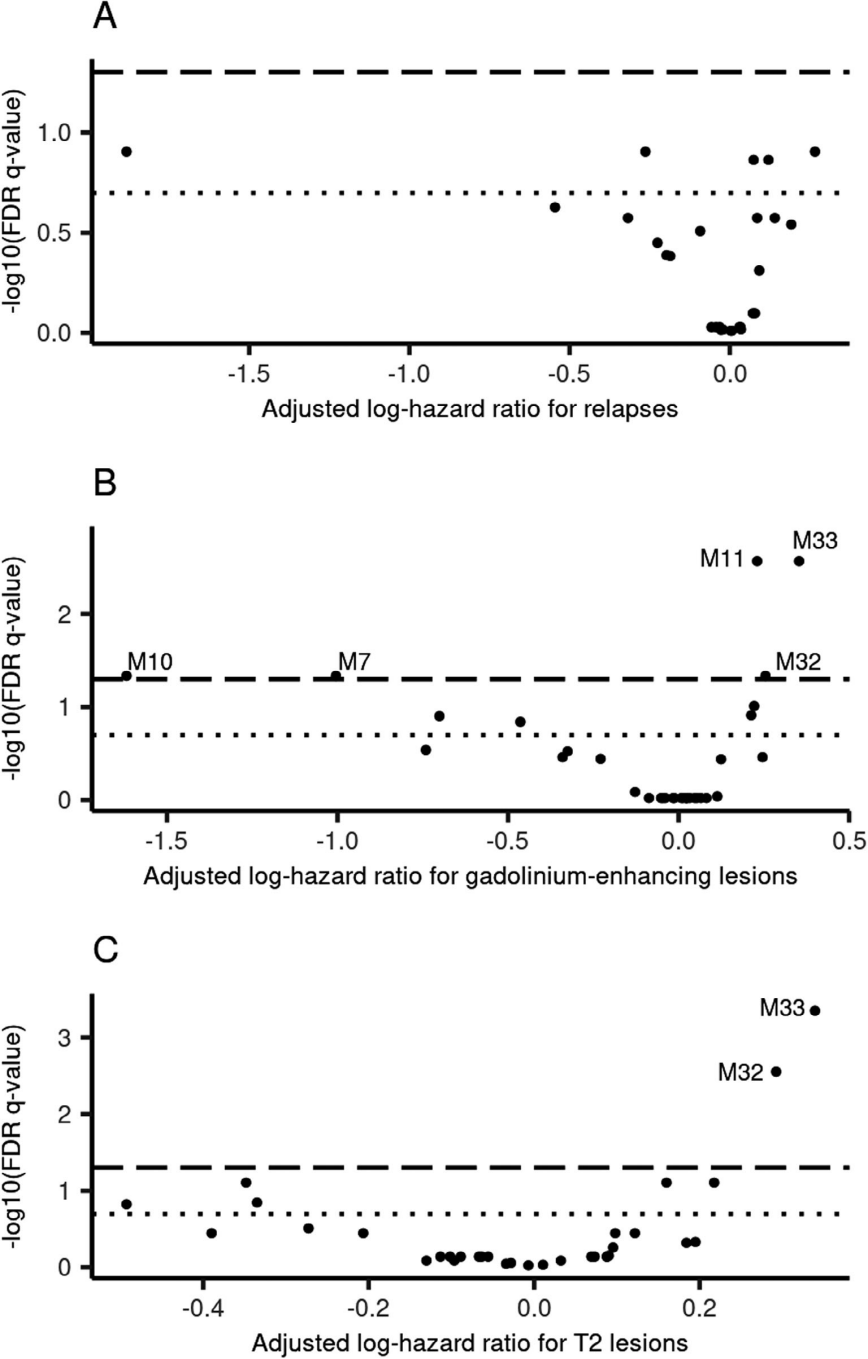
Five networks of gut microbes were significantly associated with MRI‐related multiple sclerosis activity. The long‐dashed line indicated an FDR *q* cutoff of 0.05 and the small dotted line indicated an FDR *q* cutoff of 0.2. Significant modules were labeled with their respective module name. Regression coefficients were scaled to a 0.1‐unit increase in module eigengenes because the standard 1‐unit increase would represent nearly the entire range of eigengene values. Regression models were adjusted for sex, age, and disease‐modifying therapy use. (A) Adjusted log‐hazard ratios for relapse; (B) adjusted log‐hazard ratios for new gadolinium‐enhancing lesions on MRI; and (C) adjusted log‐hazard ratios for new or enlarging T2 hyperintense lesions on MRI.

Gut microbes exist in complex, interconnected communities, so we tested the association between networks of co‐occurring microbes and each disease activity outcome. Gut microbes were classified into 33 (M1‐33) and 27 (M34‐60) modules (or clusters/networks) using the MRI and clinical cohorts, respectively (Supplementary Fig. S2). The ASVs constituting each module for MRI and clinical cohorts were shown in Supplementary Tables S2 and S3. Among the 33 modules identified from subjects within the MRI cohort, five (M7, 10, 11, 32, and 33) were significantly associated (FDR *q* < 0.05) with new gadolinium‐enhancing lesions (Fig. 6). Two of these modules were protective, where higher module values were associated with a lower hazard of new gadolinium‐enhancing lesions: M7 (HR = 0.37, 95% CI: 0.18, 0.76) and M10 (HR = 0.20, 95% CI: 0.06, 0.63). For the other three significant modules, higher module values were associated with a higher hazard of new gadolinium‐enhancing lesions: M11 (HR = 1.26, 95% CI: 1.12,1.42), M32 (HR = 1.29, 95% CI: 1.08, 1.54), and M33 (HR = 1.42, 95% CI: 1.18, 1.71). Higher M32 and M33 module values were also significantly associated with higher hazard of new or enlarging T2 hyperintense lesions (HR_M32_ = 1.34, 95% CI: 1.15 1.56; HR_M33_ = 1.41, 95% CI: 1.21, 1.64). No other modules were significantly associated with new or enlarging T2 hyperintense lesions, and no modules were significantly associated with relapse(s) (see Supplementary Tables S4 and S5 for full results). Interestingly, only one of the five ASVs shown to be individually associated with all three disease activity outcomes was a member of a significant module. This was SV_245, an unidentified member of the *Lachnospiraceae NK4A136* group (which showed a protective effect for all three disease activity outcomes) and a member of the M10 module (associated with a lower hazard for the MRI outcomes).

Several of the modules significantly associated with MRI outcomes could be mapped to the clinical modules. Notably, all four ASVs within the significant M33 MRI module were in the M56 clinical module. While not statistically significant, the effect size between the M56 module and relapse (HR_relapse_ = 1.15, 95% CI: 0.98, 1.35) was similar to the effect sizes between the M33 module and MRI outcomes (HR_Gad_ = 1.42 and HR_T2_ = 1.40). Additionally, ASVs in the significant M32 MRI module overlapped with the M38 clinical module. The effect size between the M38 module and relapse (HR_relapse_ = 1.21, 95% CI: 0.96, 1.53) was similar to the effect sizes between the M32 module and MRI outcomes (HR_Gad_ = 1.29 and HR_T2_ = 1.34).

### Predicted functional pathways from gut microbial networks were associated with multiple sclerosis

Metabolic pathways identified in each significant fecal microbial module were shown in Supplementary Tables S6‐S10. No modules were significantly associated with relapse, so pathway–relapse associations were not assessed. Metagenomic predictions indicated that modules significantly associated with MRI outcomes encoded amino acid biosynthesis pathways, including the superpathways of l‐arginine and l‐tryptophan biosynthesis (Supplementary Tables S6‐S10). Filtering pathways to those only associated with an outcome at *p* < 0.05, several pathways were specific to modules associated with a lower hazard (M10 module) or higher hazard (M32 and M33 modules) for the MRI outcomes (Supplementary Fig. S3). Notably, the superpathways of l‐tyrosine (*p* = 0.01) and l‐phenylalanine (*p* = 0.01) biosynthesis were associated with a lower hazard of MRI outcomes.

## Discussion

In this longitudinal study of subjects with pediatric‐onset MS, we identified several individual gut microbes and networks of co‐occurring microbes associated with a higher or lower hazard of clinical relapse and MRI‐related disease activity. Known functions and metagenomic predictions of these microbes suggest the important role of butyrate and amino acid biosynthesis pathways. The protective, anti‐inflammatory effects of butyrate, which have previously been observed in MS studies, provide a potential target for future microbiome interventions intended to modify disease activity in MS.

Three microbes were associated with subsequent disease activity in pediatric‐onset MS at FDR *q* < 0.2 and warrant further functional investigation. These included *Blautia stercoris* and *Christensenellaceae catabacter,* whereby having any of these bacteria nearly tripled the hazard of relapse, and *Odoribacter splanchnicus*, where having no copies increased the hazard (four times) of a new gadolinium‐enhancing lesion. In one small case–control study, higher abundance of *Blautia* was found among MS cases compared to controls.^33^ In line with our findings, a higher abundance of *Blautia* and lower abundance of *Odoribacter* have been found among individuals with active lupus disease, another autoimmune disorder, compared to controls.^34^ *Odoribacter* has also been found to be lower among individuals with cystic fibrosis, inflammatory bowel disease, and Crohn’s disease.^35^, ^36^, ^37^, ^38^ The potential benefits of *Odoribacter* were largely attributed to its production of butyrate, a short‐chain fatty acid that can help maintain gut homeostasis and suppress pro‐inflammatory cytokines.^39^, ^40^ The presence of *Odoribacter* was also identified in our study as associated with lower hazard of relapse and T2 hyperintense lesions, but results were not significant after multiple testing correction. In addition to *Odoribacter,* four gut microbes were found to be associated with all three disease activity outcomes before multiple testing correction. These included three microbes that may also be beneficial in higher amounts: *Butyricicoccus desmolans* (another butyrate‐producing microbe), an unidentified species in the genus *Lachnospiraceae NKA136 group*, and an unidentified species in the family *Ruminococcaceae*. In contrast, having any abundance of an unspecified species belonging to the *Coriobacteriales* order more than doubled the hazard of disease activity outcomes. All of these microbes had consistent effect sizes across all three MS activity outcomes. Together, these findings suggest the role of butyrate‐producing microbes in reducing the risk (hazard) of MS relapses and new/enlarging MRI lesions. This agrees with other studies that have shown oral administration of butyrate decreased demyelination in mice, serum butyric acid concentration was lower among MS cases compared to controls, and gut butyrate (assessed via metagenomics and stool metabolites) was reduced among individuals with relapsing‐remitting MS.^41^, ^42^, ^43^

Individual microbes are unlikely to work independently, and for the first time, unsupervised machine learning has identified networks (or modules/clusters) of co‐occurring gut microbes associated with disease activity outcomes in MS. We identified five networks of co‐occurring gut microbes associated with an altered risk of new gadolinium‐enhancing lesions, of which two were also associated with T2 hyperintense lesions. Across these five modules, pathways involving aromatic amino acid biosynthesis were predicted to be enriched. Namely phenylalanine and tyrosine biosynthesis pathways were enriched in the M10 module (a module significantly associated with a lower hazard of MRI‐related outcomes), while tryptophan was enriched in the M10, M32, and M33 modules (M32 and 33 were associated with a higher hazard of MRI‐related outcomes). This relationship of tryptophan with both increased and decreased risk may relate to differences in the expression of genes in these microbial modules or their differential catabolism to bioactive metabolic products, for example, kynurenine. Tryptophan, specifically, has been identified as a modulator of the central nervous system inflammation and associated with MS risk and course.^44^, ^45^, ^46^ Interestingly, serum metabolite studies of MS activity have identified shifts in aromatic amino acid metabolism among individuals with worse disease activity.^47^, ^48^ Our findings suggest that networks of gut microbes associated with MS activity may contribute to the concentration of amino acids, specifically aromatic amino acids that serve as potent CNS and immunomodulatory signaling molecules.

There are several notable strengths of this study. We were able to include individuals with MS shortly after disease onset and follow them prospectively. This captured clinically relevant relapses and MRI data, the latter of which are considered highly sensitive and useful when assessing changes in disease activity over time. Further, our participants were well characterized and were either not using a DMT or using drugs with low effectiveness (in terms of relapse prevention) at baseline. Pediatric‐onset cases are useful because it allows for the examination of disease processes much closer to biological onset compared to adults in individuals with very few confounding comorbidities.^13^, ^49^, ^50^, ^51^ While the gut microbiome does undergo significant changes in very early childhood, it is relatively stable in adolescence, with functional capacity similar to adults.^52^ Because our sample was almost entirely enrolled as adolescents, this potential source of variation was limited.

While a pediatric‐onset cohort represents a unique opportunity for studying modifiers of MS, its rarity limited our sample size and ability to account for other potential confounders or modifiers, such as study site, race, ethnicity, body mass index, diet, vitamin D status, and specific DMTs. The small sample size and large number of multiple tests also made it particularly challenging for a result to achieve statistical significance, despite potential biological significance. Because this study is the first of its kind, we reported individual ASV findings with FDR *q* < 0.2 despite not reaching statistical significance (FDR *q* < 0.05). It is possible these may be false positive findings, and should thus be conservatively interpreted. They should be considered candidates for future functional studies and hypotheses and replicated in future work. Another limitation was that all MRI scans were performed without a centralized or standardized imaging protocol and the timing of scans was not at predetermined intervals (as part of routine clinical practice). Finally, metagenomic predictions cannot be interpreted as true functions or pathways. However, our findings warrant further investigation, including microbes that influence butyrate and amino acid synthesis pathways. We do not have metabolomic or metagenome data to confirm predicted findings, which should be the focus of future work. Additionally, it would be useful for future studies to collect stool samples repeatedly over time to assess how changes in gut composition due to treatment, diet, and other factors might be associated with relapses and MRI outcomes over time.

In summary, we identified several individual gut microbes and networks of co‐occurring microbes that were associated with an altered risk of clinical relapse and activity on brain MRI among pediatric‐onset MS patients. Known functions and metagenomic predictions of these organisms suggest the roles of butyrate and amino acid biosynthesis as potential modifiers of MS activity. Further research is needed to confirm the functional and clinical implications of these findings, so personalized microbiome interventions may be designed to decrease MS activity.

## Author Contributions

E.W. and S.V.L. contributed to the conception and design of the study, acquisition and analysis of data, and drafting a significant portion of the manuscript or figures. M.K.H., K.M., J.G., J.N., Y.W., M.G., L.B., B.WG., A.W., M.R., JM.T., L.K., A.B., T.C.C, J.R., J.H., X.S, H.T., and L.F.B contributed to the acquisition and analysis of data and drafting a significant portion of the manuscript or figures. D.F. and K.F. contributed to the acquisition and analysis of data. S.M., M.R., and T.C. contributed by drafting a significant portion of the manuscript or figures.

## Conflict of Interest

The authors report no competing interests.

## Supporting information

**Supplementary Table S1.** Results of time‐to‐event analyses for each amplicon sequence variant (ASV) and pediatric‐onset multiple sclerosis activity outcome.
**Supplementary Table S2.** Amplicon sequence variant (ASV) membership for wgcna modules obtained from MRI cohort (n = 46).
**Supplementary Table S3.** Amplicon sequence variant (ASV) membership for wgcna modules obtained from clinical cohort (n = 54).
**Supplementary Table S4.** Results of recurrent time‐to‐event analyses for each module (obtained from MRI cohort) and MRI outcomes (new gadolinium‐enhancing lesions and new or enlarging T2 lesions). Hazard ratios (HRs) adjusted for sex, age, and time‐varying disease‐modifying therapy use and 95% confidence intervals (CIs). HRs and 95% CIs are scaled to 0.1‐unit increases in module eigengenes.
**Supplementary Table S5.** Results of recurrent time‐to‐event analyses for each module (obtained from clinical cohort) and relapse(s). Hazard ratios (HRs) adjusted for sex, age, and time‐varying disease‐modifying therapy use and 95% confidence intervals (CIs). HRs and 95% CIs are scaled to 0.1‐unit increases in module eigengenes.
**Supplementary Table S6.** Supplementary Table 6. PICRUSt2 predicted 137 pathways within the M7 module. Hazard ratios (HRs) and 95% confidence intervals (CIs) were presented for pathways in at least 20% of samples for new gadolinium‐enhancing lesions. The M7 module was not associated with relapse or T2 lesions, so pathway associations were not estimated for these outcomes.
**Supplementary Table S7.** PICRUSt2 predicted 209 pathways within the M10 module. Hazard ratios (HRs) and 95% confidence intervals (CIs) were presented for pathways in at least 20% of samples for new gadolinium‐enhancing lesions. The M10 module was not associated with relapse or T2 lesions, so pathway associations were not estimated for these outcomes.
**Supplementary Table S8.** PICRUSt2 predicted 122 pathways within the M11 module. Hazard ratios (HRs) and 95% confidence intervals (CIs) were presented for pathways in at least 20% of samples for new gadolinium‐enhancing lesions. The M11 module was not associated with relapse or T2 lesions, so pathway associations were not estimated for these outcomes.
**Supplementary Table S9.** PICRUSt2 predicted 168 pathways within the M32 module. Hazard ratios (HRs) and 95% confidence intervals (CIs) were presented for pathways in at least 20% of samples for MRI outcomes. The M32 module was not associated with relapse, so pathway associations with relapse were not estimated. Results are sorted by gadolinium‐enhancing FDR *q* and HR and then T2 lesion FDR *q* and HR.
**Supplementary Table S10.** PICRUSt2 predicted 131 pathways within the M33 module. Hazard ratios (HRs) and 95% confidence intervals (CIs) were presented for pathways in at least 20% of samples. The M33 module was not associated with relapse, so pathway associations with relapse were not estimated. Results are sorted by gadolinium‐enhancing FDR *q* and HR and then T2 lesion FDR *q* and HR.

**Supplementary Figure S1.** Total proportion of abundance of microbes (by class) according to baseline disease‐modifying therapy status. The proportion of individuals ASVs belonging to a particular taxonomic class did not significantly differ by baseline DMT use categories (none, glatiramer acetate, interferon beta, or other DMT) except for Melainabacteria (*p* = 0.0002) and Verrucomicrobiae (*p* = 0.049). *p*‐values determined from F‐test.
**Supplementary Figure S2.** Weighted genetic correlation network analysis identified 33 modules of co‐occurring microbes for the MRI cohort and 27 modules for the clinical cohort. (A and B) Bacterial taxa dendrogram and module names (colors) for MRI and clinical cohorts, respectively. Each node was an ASV. Taxa that co‐occur were positioned closer together and the module for which an ASV was a member was plotted in a vertical band below. (C and D) Clustering of module eigenvalues for MRI and clinical cohorts, respectively, with corresponding names (number and color).
**Supplementary Figure S3.** Pathways predicted to be associated with significant gut microbial modules from PICRUSt2. Each row was a MetaCyc pathway that was associated with a pediatric‐onset multiple sclerosis outcome at *p* < 0.05 in at least one of the five significant microbial modules (M7, 10, 22, 32, 33). Colors indicated the magnitude of hazard ratios, adjusted for sex, age, and disease‐modifying therapy use, which represented the association between a pathway and pediatric‐onset MS activity outcome, per module (columns). Hazard ratios were only estimated for disease activity outcomes previously identified as associated with a respective module. Gray indicated the module‐specific pathway‐outcome association had *p* ≥ 0.05. Hazard ratios were estimated for pathways present in at least 20% of the respective cohort and module. No pathways within the M7 or M11 modules were significant, so were not shown. Abbreviations: Gad, gadolinium; MS, multiple sclerosis.
