# Supplementary material for: Gut microbiome is associated with multiple sclerosis activity in children
Source: Ann Clin Transl Neurol. 2021 Aug 19;8(9):1867–83. doi: 10.1002/acn3.51441 (PMC8419410; doi:10.1002/acn3.51441)
Supplement: Supplementary file 2 — Supplementary Figure S1. Total proportion of abundance of microbes (by class) according to baseline disease‐modifying therapy status. The proportion of individuals ASVs belonging to a particular taxonomic class did not significantly differ by baseline DMT use categories (none, glatiramer acetate, interferon beta, or other DMT) except for Melainabacteria (p = 0.0002) and Verrucomicrobiae (p = 0.049). p‐values determined from F‐test. Supplementary Figure S2. Weighted genetic correlation network analysis identified 33 modules of co‐occurring microbes for the MRI cohort and 27 modules for the clinical cohort. (A and B) Bacterial taxa dendrogram and module names (colors) for MRI and clinical cohorts, respectively. Each node was an ASV. Taxa that co‐occur were positioned closer together and the module for which an ASV was a member was plotted in a vertical band below. (C and D) Clustering of module eigenvalues for MRI and clinical cohorts, respectively, with corresponding names (number and color). Supplementary Figure S3. Pathways predicted to be associated with significant gut microbial modules from PICRUSt2. Each row was a MetaCyc pathway that was associated with a pediatric‐onset multiple sclerosis outcome at p < 0.05 in at least one of the five significant microbial modules (M7, 10, 22, 32, 33). Colors indicated the magnitude of hazard ratios, adjusted for sex, age, and disease‐modifying therapy use, which represented the association between a pathway and pediatric‐onset MS activity outcome, per module (columns). Hazard ratios were only estimated for disease activity outcomes previously identified as associated with a respective module. Gray indicated the module‐specific pathway‐outcome association had p ≥ 0.05. Hazard ratios were estimated for pathways present in at least 20% of the respective cohort and module. No pathways within the M7 or M11 modules were significant, so were not shown. Abbreviations: Gad, gadolinium; MS, multiple sclerosis. [file ACN3-8-1867-s001.pdf]

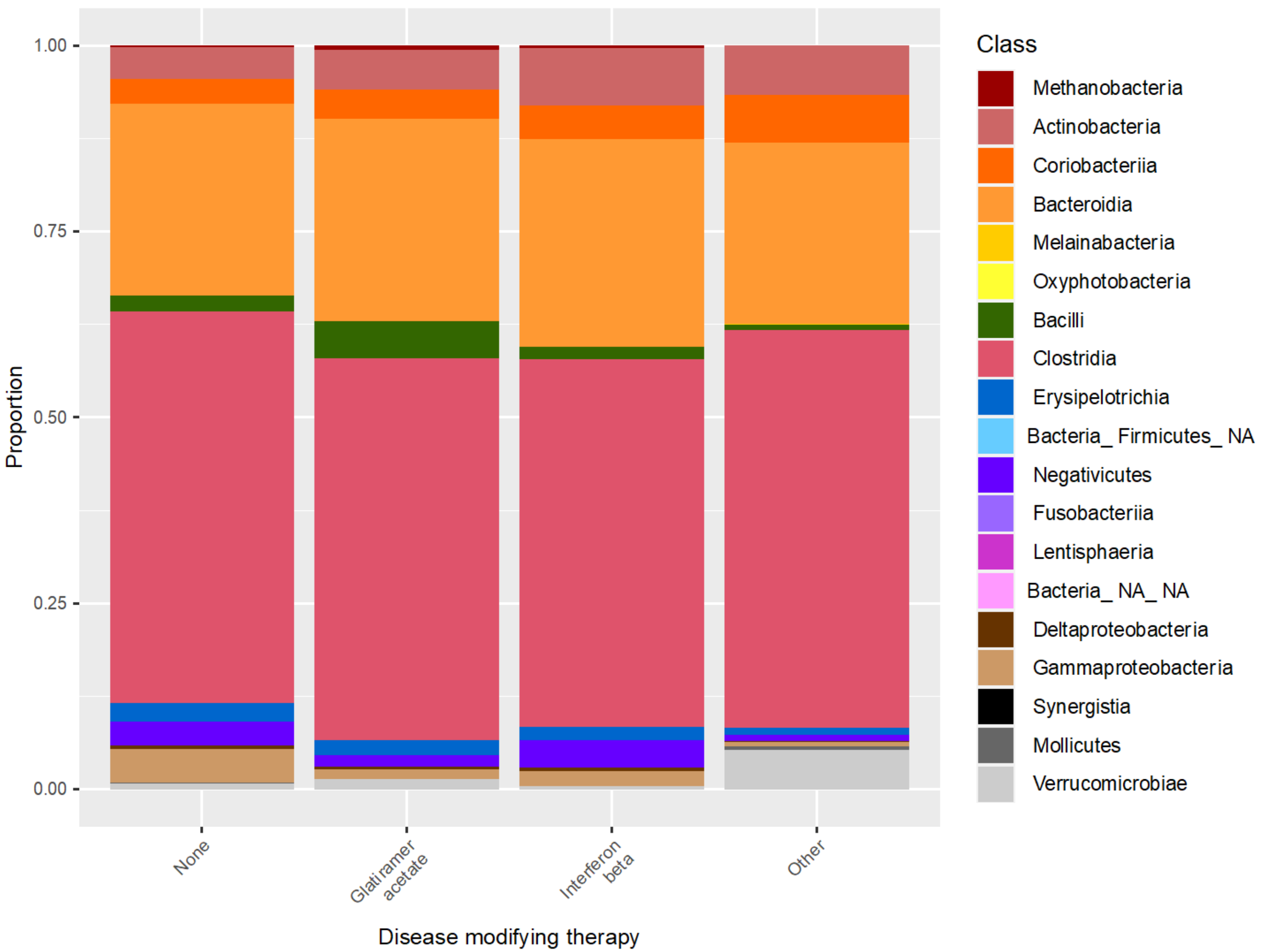

Supplementary Figure 1. Total proportion of abundance of microbes (by class) according to baseline disease modifying therapy status. The proportion of individuals ASVs belonging to a particular taxonomic class did not significantly differ by baseline DMT use categories (none, glatiramer acetate, interferon beta, or other DMT) except for Melainabacteria (p=0.0002) and Verrucomicrobiae (p=0.049). P-values determined from F-test.

A Gut microbe dendrogram and module colors for MRI cohort

B Gut microbe dendrogram and module colors for clinical cohort

C Eigengene dendrogram for MRI cohort

D Eigengene dendrogram for clinical cohort

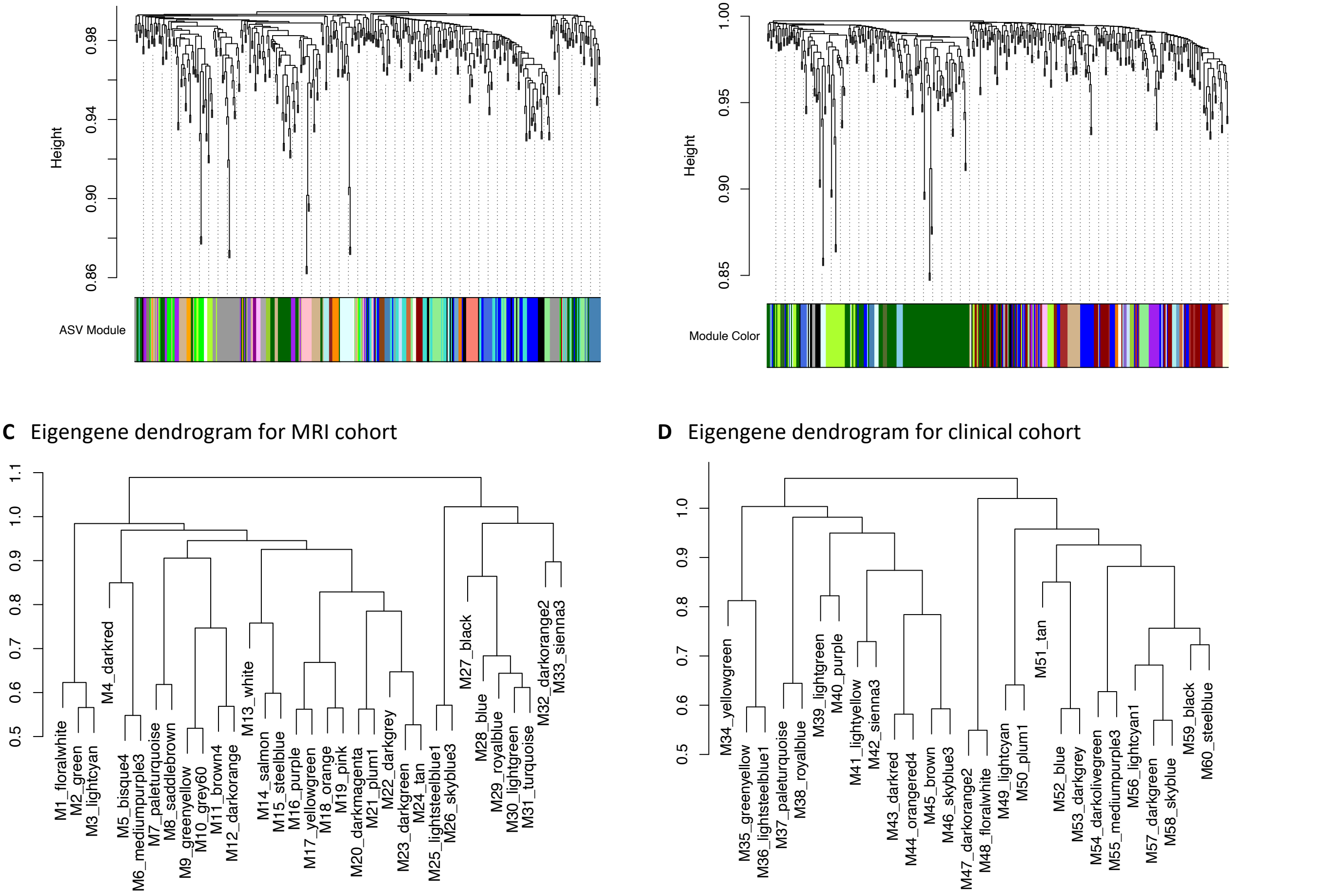

Supplementary Figure 2 Weighted genetic correlation network analysis identified 33 modules of co-occurring microbes for the MRI cohort and 27 modules for the clinical cohort. **(A and B)** Bacterial taxa dendrogram and module names (colors) for MRI and clinical cohorts, respectively. Each node was an ASV. Taxa that co-occur were positioned closer together and the module for which an ASV was a member was plotted in a vertical band below. **(C and D)** Clustering of module eigenvalues for MRI and clinical cohorts, respectively, with corresponding names (number and color).

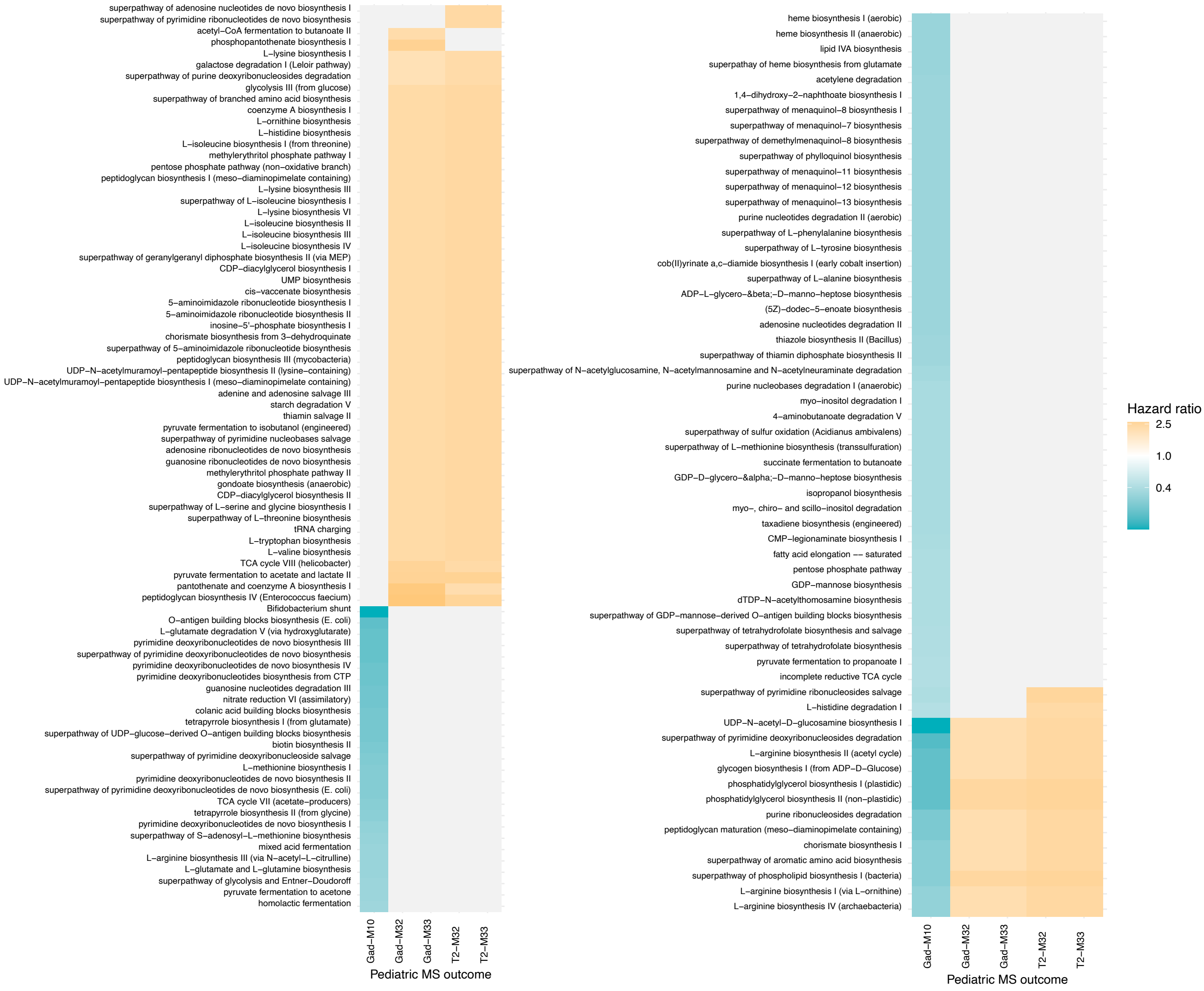

Supplementary Figure 3. Pathways predicted to be associated with significant gut microbial modules from PICRUSt2. Each row was a MetaCyc pathway that was associated with a pediatric-onset multiple sclerosis outcome at $p<0.05$ in at least one of the five significant microbial modules (M7, 10, 22, 32, 33). Colors indicated the magnitude of hazard ratios, adjusted for sex, age, and disease modifying therapy use, which represented the association between a pathway and pediatric-onset MS activity outcome, per module (columns). Hazard ratios were only estimated for disease activity outcomes previously identified as associated with a respective module. Grey indicated the module-specific pathway-outcome association had $p\geq0.05$. Hazard ratios were estimated for pathways present in at least 20% of the respective cohort and module. No pathways within the M7 or M11 modules were significant, so were not shown. Abbreviations: Gad = gadolinium, MS = multiple sclerosis.
